# Pregnancy wastage among HIV infected women in a high HIV prevalence district of India

**DOI:** 10.1186/s12889-015-1965-1

**Published:** 2015-07-02

**Authors:** Shiva S. Halli, C.G. Hussain Khan, Iqbal Shah, Reynold Washington, Shajy Isac, Stephen Moses, James F. Blanchard

**Affiliations:** Centre for Global Public Health, Department of Community Health Sciences, College of Medicine, Faculty of Health Sciences, University of Manitoba, 771 McDermot Avenue, Winnipeg, Manitoba R3E 0T6 Canada; Department of Anthropology, Karnataka University, Dharwad, Karnataka 586003 India; Chemin de Malvand, Chambesy, 12C 1292 Switzerland; Karnataka Health Promotion Trust, 1-4, IT Park, 5th Floor, Rajajinagar Industrial Area, Bangalore, Rajajinagar 560044 India; Centre for Global Public Health, Department of Community Health Sciences, College of Medicine, Faculty of Health Sciences, University of Manitoba, 771 McDermot Avenue, Winnipeg, Manitoba R3E 0T6 Canada; Department of Community Health Sciences, College of Medicine, Faculty of Health Sciences, University of Manitoba, 771 McDermot Avenue, Winnipeg, Manitoba R3E 0T6 Canada

**Keywords:** HIV, Pregnancy wastages, Abortions, Still births, Early age at marriage

## Abstract

**Background:**

Bagalkot district in Karnataka state is one of the highest HIV prevalence districts in India. A large proportion of the girls also marry at early age in the district and negative pregnancy outcomes among the HIV positive women likely to have large pregnancy wastages. Therefore, this study examined the pregnancy wastages and the associated factors among HIV positive women in a high prevalent district in India.

**Methods:**

We used data from a cross-sectional survey conducted recently among randomly selected currently married HIV positive women, 15–29 years of age, in one of the high HIV prevalence districts in India. The study used the experience of reported pregnancy wastage as an outcome variable, and both bi-variate and multivariate logistic regression analyses were carried out to understand the factors associated with the pregnancy wastage among HIV infected women.

**Results:**

Overall, 17 % of the respondents reported pregnancy wastage, of which 81 % were due to spontaneous abortions. Respondents who became pregnant since testing HIV positive reported significantly higher level of pregnancy wastage as compared to those were pregnant before they were tested for HIV. (AOR = 1.9; p = 0.00). While a positive association between duration of marriage and pregnancy wastage was noticed (AOR = 7.4; p = 0.01), there was a negative association between number of living children and pregnancy wastage (AOR = 0.24; p = 0.00). Living in a joint family was associated with increased reporting of pregnancy wastage as compared to those living in nuclear families (AOR = 1.7; p = 0.03).

**Conclusions:**

HIV prevention and care programs need to consider the reproductive health needs of HIV infected married women as a priority area since large proportion of these women reported negative pregnancy outcomes. There is also a need to explore ways to raise the age at marriage in order to stop women getting married before the legal age at marriage.

## Background

Globally, it is estimated that there were 35.3 million people living with HIV and 2.3 million new infections in 2012 [1]. Although the burden of the epidemic varies considerably between countries and regions, it is estimated that 0.8 % of adults aged 15–49 years worldwide are living with HIV [1]. The HIV/AIDS epidemic remains a serious public health challenge, especially among women of childbearing age, who make up 46 % of the global HIV burden. The UNAIDS Global Report (2013) also highlights that women constitute 52 % of all HIV infections in low- and middle-income countries [1]. HIV infected women are vulnerable to various reproductive health issues. Spontaneous abortion and stillbirths appear to be more common among HIV-infected women [2, 3]. In Western countries, HIV positive women, have higher rates of induced abortion [4], but it is difficult to tell whether this is also true in Asia. Medical termination of Pregnancy is legalised in India, but rural young and unmarried women frequently access unqualified practitioners who conduct abortions in unsafe settings [5].

Data from Zaire (now Democratic Republic of Congo) suggest that there is not much difference in induced abortions among HIV-infected women compared to non-infected in sub-Saharan Africa [6]. Evidence suggests that women presenting with spontaneous abortion had a significantly higher rate of HIV infection than those presenting for antenatal care or delivery [7]. Moreover, the same study also noted that HIV infected women had a much higher rate of recent history of fetal wastage (late abortions, neonatal deaths, premature delivery) than the non-infected group.

India is heterogeneous in socio-demographic and economic contexts with low fertility behaviours in a number of southern states as compared to northern states. Data from National Family Health Survey (NFHS-3) suggest that overall 14.4 % of the ever married women ever experienced a non-live births (still births, abortions and miscarriages) [8]. The data further showed that a lower percent of 15–29 years old ever married women reported experiencing a non-live birth (10.1 %). Regional differentials were evident as far as the reported non-live birth in India with some states reporting over 20 % and 7.9 % women aged 15–49 years in Karnataka reported a non-live birth.

HIV epidemic in India is driven by heterosexual behaviours with HIV prevalence among the general population is estimated at 0.36 % and an estimated people living with HIV (PLHIV) of 2.5 million in India [9].

Karnataka state in India is considered a high HIV prevalence state, with an average of 0.69 % of adults aged 15–49 years estimated to be HIV positive [8]. The HIV positivity rate among the 776,185 pregnant women tested during January-December 2010 in the state was 0.36 % [10]. The district of Bagalkot, in northern Karnataka, is one of the highest prevalence districts in the country, with a HIV prevalence of 2.6 % among the general population in 2009, significantly higher in rural areas than urban areas [11]. Bagalkot (1.26 %), was also the only district in the state that had a HIV positivity rate of greater than 1 % among pregnant women tested in 2010. The district has also reported relatively high levels of neonatal and child mortality compared to many other districts in the state [12].

Few studies attempted to understand the pregnancy outcomes among PLHIVs in India. A study conducted among Prevention of Parent to Child Transmission (PPTCT) attendees in Kadapa district of Andhra Pradesh, India found that low birth weight is an observed complication in HIV infected [13]. Further, negative pregnancy outcomes (abortions and high still births) are significantly high among HIV positive women [7, 13]. Kumar et al. [13] studied pregnancy outcomes among HIV positive women, based on a cohort of pregnant women attending HIV testing centres over a period of one year in India. Although the study recruited 4112 pregnant women, only 56 were HIV positive, so the relationship between HIV and pregnancy wastage was based on a small sample. Considering India’s socio-demographic heterogeneity and differentials of negative pregnancy outcomes in the country, it is important to understand the pregnancy outcomes among PLHIV. Further, only two studies were available in the country, which discussed on pregnancy outcomes among PLHIVs using a small purposive sample. Therefore, in this paper, we examine the effect of HIV among pregnant women in a high HIV prevalent district, Bagalkot, particularly on the outcomes of abortions and still births, from a large random sample of HIV positive women.

## Methods

### Sampling design

The sample size for the study was decided based on 5 % marginal of error and 80 % power with 95 % confidence level. Considering the measure of interest, namely couple protection rate of 50 %, the following statistical formula was used.

$$ \left(\mathrm{Z}2\kern0.5em *\kern0.5em \mathrm{p}\mathrm{q}\right)/{\mathrm{d}}^2; $$

Using the above formula, the required sample size is 384. However, the sample was increased accounting a design effect of 1.7 as well non-response of 10 % m the required sample size was around 720.

We recruited a total of 720 young (15–29 years) HIV positive currently married women. Of the 720 participants recruited, 633 participated (87 % response), and were administered a structured, pre-tested questionnaire in a face-to-face interview. The sample size for the study was restricted to married women, as in the context of Karnataka, childbearing is an option mainly for married women.

The *Jeevan Jyothi* community-based organization (CBO) in Bagalkot district is one of the earliest CBOs formed for HIV positive people in Karnataka state. The *Jeevan Jyothi* has established a drop-in centre (DIC) for HIV positive people in the district to access various HIV related services. Most of the (approximately 4,000) women registered with the DIC consented to be contacted, and about 2,000 were in the age group 15–29 years and were included in the sampling frame. The required sample of respondents (720) was then selected using a stratified random sampling approach, where age was considered as a stratification variable. Currently married women were stratified into 3 age groups: 15–19 years, 20–24 years and 25–29 years, and then the required number of respondents were randomly selected from each strata proportionately.

### Data collection

The field team included, 6 female interviewers and 1 supervisor. All the field investigators were at least graduates with significant field experience in collecting data. All the field team members were given one week intensive training, which included, class room lectures, orientation on questionnaire, mock interviews, visit to Jeevan Jyoti and field practices. In addition, they were trained on conducting interviews and ethics of researches. A special training was given to the supervisor in data quality control, use of monitoring tools, preparation of progress report, inter-personal communication with staff and others etc. The field work was carried out during July-August 2012.

The study used a structured interview questionnaire which was administered to each selected participant. The study questionnaire included information on fertility preferences of Women Living with HIV/AIDS, socio-economic and demographic characteristics, reproductive behaviour and contraceptive use, fertility behaviour after having HIV/AIDS, reproductive and sexual health etc.

### Data analysis

The study used SPSS 22.0 statistical software, and bivariate as well as multivariate analyses were carried out. Each respondent in the survey was asked “if she ever had pregnancy that ended in still birth, spontaneous or induced abortion?”. If she had experienced any, then questions were also asked how many still births, induced and spontaneous abortions. The present study included any women reported experience of still births or abortions. We compared reported pregnancy wastages among two groups of women, the group of women who became pregnant after their HIV positive status vs. the group who did not become pregnant after their HIV positive status but had pregnancies prior to HIV positive status. Multivariate logistic regression models were used to understand the correlates of reported pregnancy wastage, incorporating a set of independent variables, including place of usual residence, literacy level, occupation, age at living with current husband, duration of marriage, literacy of husband, occupation of husband, caste, type of house, type of family, monthly household income, number of living children and duration since HIV tested.

### Ethical considerations

Ethical clearance was obtained from the World Health Organization’s Research Ethics Review Committee, the Institutional Ethical Review Board of St John’s Medical College, Bangalore, and the Health Research Ethics Board of the University of Manitoba, Winnipeg, Canada.

## Results

### Profile of women living with HIV

Of the 633 respondents interviewed, 27 % were less than 25 years of age, with a mean age of about 26 years. Over 71 % were from rural areas, about half were illiterate and 45 % of the respondents’ husbands were illiterate as well. Most of the respondents were Hindu (95 %), 42 % belonged to scheduled castes or scheduled tribes (SC/ST); and another 28 % belonged to “other backward classes” (OBC). About 85 % of respondents were engaged in labour or household work, and almost all of the respondents were currently living with their husbands and had been married only once.

Over one-fourth (28 %) of the women started living with their current husbands before the age of 15 years, and as high as 73 % of respondents started living with their current husbands before the legal age of marriage of 18 years. About 83 % of respondents had an age gap between respondent and husband of over 5 years. The mean age of the respondents was 25.6 years, vs. 32.5 years among their husbands, indicating a mean age gap between respondent and husband of about 7 years. Approximately 9 % respondents had been married for less than 5 years, 34 % and 57 % respectively had been married for 5–9 years and 10+ years with median duration of marriage of 10 years. While most husbands were engaged in cultivation or manual labour, 26 % worked in a business or were salaried employees.

The mean monthly household income was about Rs 7,380 (US $120), and only one-fourth of the respondents had a monthly household income above Rs 10,000 (US $167). Two-fifths of households (43 %) had a monthly household income between Rs 5,000 - Rs 9,999 (US $83-167), 23 % had a monthly income between Rs 3,000-Rs 4,999 (US $50-83) and 9 % had a monthly income less than Rs 3,000 (<US $50). About 91 % of respondents lived in their own house. The households were poorly maintained, with about 39 % of respondents living in thatched or mud- roofed households. Over three-quarters (79 %) of respondents were living in a nuclear family, and on an average a respondent had 1.82 living children.

While about one-fourth of the respondents had recently tested HIV positive (during the past 2 years), 49 % and 25 % respectively were tested HIV positive 2–3 years and 4+ years prior to the survey, with a mean duration since testing HIV positive of 2.61 years with a standard deviation of 1.92. Over one-third (37 %) of the respondents had become pregnant after testing HIV positive.

### Levels of pregnancy wastage among WLHAs by characteristics

**Fig. 1:**
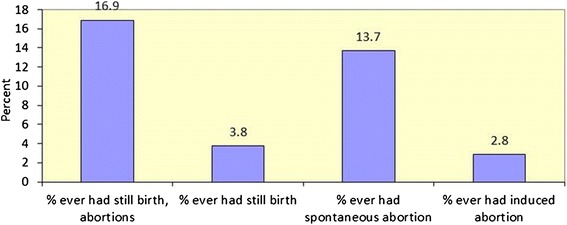
Percent HIV positive women experienced pregnancy wastages

The study examined the differential levels of reported pregnancy wastages by characteristics of the respondents and is presented in Fig. 1 and Table 1. Overall, 17 % of the respondents reported ever having reported experienced pregnancy wastage, including stillbirths, induced and spontaneous abortions (Fig. 1). This included 3.8 % with stillbirths, 13.7 % with spontaneous abortions and 2.8 % with induced abortions.

**Table 1:** Percent of HIV positive women experiencing of pregnancy wastage by characteristics

Characteristics		Number	% ever had still birth or abortions	Odds Ratio	95 % Confidence Interval		p
					Lower	Upper	
Total		633	16.9				
Age	<25 years	168	18.5	1.00			
	25+ years	465	16.3	0.86	0.54	1.37	0.53
Place of usual residence	Urban	179	16.2	1.00			
	Rural	454	17.2	1.07	0.67	1.71	0.77
Literacy level	Literate	327	16.5	1.00			
	Illiterate	306	17.3	1.06	0.70	1.61	0.79
Occupation	Cultivator	50	18.0	1.00			
	Labour	259	17.8	0.98	0.45	2.16	0.97
	Household work	279	16.5	0.90	0.41	1.98	0.79
	Others	45	13.3	0.70	0.23	2.15	0.53
Travel due to work	Yes	57	5.3	1.00			
	No	576	18.1	3.97	1.22	12.93	0.02
Age at living with current husband	<15 years	175	21.1	1.00			
	15-17 years	287	17.4	0.78	0.49	1.26	0.32
	18+ years	170	11.2	0.46	0.26	0.85	0.01
Durations of marriage	<5 years	57	7.0	1.00			
	5-9 years	216	17.1	2.74	0.93	8.03	0.07
	10+ years	359	18.1	2.93	1.02	8.38	0.05
Literacy of husband	Literate	348	15.2	1.00			
	Illiterate	285	18.9	1.30	0.86	1.97	0.22
Occupation of husband	Cultivator	191	16.2	1.00			
	Labour	251	17.9	1.13	0.68	1.86	0.64
	Business/Salaried	167	14.4	0.87	0.49	1.55	0.63
	Others	24	29.2	2.13	0.81	5.55	0.12
Caste	SC/ST	267	15.4	1.00			
	OBC	177	19.8	1.36	0.83	2.23	0.23
	Others	189	16.4	1.08	0.65	1.80	0.76
Type of house	Thatched/mud roof	248	19.8	1.00			
	Others	385	15.1	0.72	0.47	1.10	0.13
Monthly HH income	<Rs 3000	58	24.1	1.00			
	Rs 3000-4999	145	20.0	0.79	0.38	1.62	0.52
	Rs 5000-9999	275	14.9	0.55	0.28	1.09	0.09
	Rs 10000+	155	14.8	0.55	0.26	1.16	0.11
Type of family	Nuclear	497	15.3	1.00			
	Others	136	22.8	1.64	1.02	2.61	0.04
Total number of living children	0	92	23.9	1.00			
	1-2	366	17.5	0.67	0.39	1.17	0.16
	3+	175	12.0	0.43	0.22	0.84	0.01
Duration since tested HIV-years	<2 year	165	15.2	1.00			
	2-3 years	311	18.0	1.23	0.74	2.06	0.43
	4+ years	157	16.6	1.11	0.61	2.02	0.73
Pregnant since tested positive	No	400	14.3	1.00			
			21.5				
	Yes	233		1.64	1.08	2.50	0.02

Table 1 presents the proportion of women who reported pregnancy wastage by socio-demographic characteristics. Women living with HIV reported as experienced pregnancy wastages more or less at the same level by age of the respondents, by rural and urban residence and by education status of the respondents. Those women who did not travel for work were more likely to report pregnancy wastage (18.1 %) compared to women who travelled for work (5.3 %; OR = 3.97; CI = 1.22-12.93; p = 0.02). Younger age at living with the current husband is associated with the higher reported pregnancy wastage, 21 % of the women who started living with their husbands before the age of 15 years reported a pregnancy wastage compared to 17 % of women living with husbands between 15–17 years (OR = 0.79; CI = 0.49-1.26; p = 0.32) and 11 % of women living with husbands above the age of 18 years (OR = 0.47; CI = 0.26-0.85; p = 0.01).

Those respondents with longer than 10 years of marriage were more likely to report pregnancy wastage as compared to those married for less than 5 years (OR = 2.93; CI = 1.02-8.38; p = 0.04). While many other factors such as age gap between husband and respondent, caste, type of house and monthly household incomes were not associated with pregnancy wastage, type of family and number of living children were associated with pregnancy wastage. Those living in joint family and having fewer living children were more likely to have reported as pregnancy wastage (p < 0.05) as compared to their counterparts. Although duration since having tested HIV positive was not significantly associated with women reporting pregnancy wastage, those who became pregnant since testing HIV positive were significantly more likely to report pregnancy wastage (OR = 1.64; CI = 1.08-2.50; p = 0.02).

### Associated factors of reported pregnancy wastage

Multivariate logistics model was used to understand the determinants of reported pregnancy wastages. The adjusted odds ratios and P values are presented in Table 2 only for the variables which were significant (P < 0.05) in bivariate models. Duration of marriage, type of family, number of living children, and whether pregnant since testing HIV positive, were all associated with reported pregnancy wastage. Women who became pregnant after testing HIV positive had almost double the risk of reporting pregnancy wastage (AOR = 1.96; CI = 1.2-3.2; p = 0.00) even after controlling other variables. Increased duration of marriage increased the risk of reporting pregnancy wastage. A woman with 5–9 years duration of marriage had an increased reporting of pregnancy wastage as compared to women with less than 5 years of duration of marriage (AOR = 4.23; CI = 1.16-15.45; p = 0.02). Similarly, women having 10+ years of duration of marriage had further increased risk as compared to women with less than 5 years of marriage duration (AOR = 7.45; CI = 1.46-38.03; p = 0.01.

**Table 2:** Unadjusted and adjusted odd ratios of factors associated with pregnancy wastage

		Unadjusted				Adjusted			
		Odds Ratio	95 % C.I. for Odds Ratio		P	Odds Ratio	95 % C.I. for Odds Ratio		P
			Lower	Upper			Lower	Upper	
Travel due to work	No	1.00				1.00			
	Yes	0.25	0.07	0.82	0.02	0.30	0.08	1.09	0.06
Age at living with husband	<15	1.00				1.00			
	15-17	0.78	0.49	1.26	0.32	0.85	0.50	1.45	0.56
	18+	0.46	0.25	0.85	0.01	0.72	0.29	1.77	0.48
Age gap between husband and respondent	<5	1.00				1.00			
	5-9	1.30	0.70	2.42	0.39	1.36	0.65	2.82	0.40
	10+	2.08	0.99	4.35	0.05	2.15	0.75	6.11	0.14
Duration of marriage	<5	1.00				1.00			
	5-9	2.73	0.93	8.03	0.06	4.23	1.15	15.46	0.02
	10+	2.92	1.02	8.38	0.04	7.45	1.46	38.03	0.01
Type of family	Nuclear	1.00				1.00			
	Other	1.63	1.02	2.61	0.04	1.77	1.03	3.04	0.03
Number of living children	0	1.00				1.00			
	1-2	0.67	0.38	1.16	0.16	0.37	0.19	0.71	0.00
	3+	0.43	0.22	0.84	0.01	0.24	0.11	0.53	0.00
Pregnant since tested HIV	No	1.00				1.00			
	Yes	1.64	1.08	2.50	0.02	1.96	1.20	3.20	0.00
Had history of child death	No	1.00				1.00			
	Yes	1.62	1.04	2.53	0.03	1.33	0.80	2.20	0.25

Type of family was associated with reported pregnancy wastage. HIV positive women in a joint family were more likely to report pregnancy wastage compared to those women in a nuclear family (AOR = 1.77; CI = 1.04-3.05; p = 0.03). Women with higher numbers of living children (3+ children) were less likely to report pregnancy wastage, compared to women with no living children (AOR = 0.24; CI = 0.11-0.53; p = 0.00).

## Discussion

DLHS (IIPS, 2007–08) showed that in the state of Karnataka and in Bagalkot district, about 4 % of all pregnancies among the general population resulted in spontaneous abortions. However, our study showed that as high as 14 % of our sample of currently married HIV infected women had ever reported experiencing spontaneous abortions, and about 4 % had reported experiencing stillbirths, indicating a very high level of pregnancy wastage. The most conspicuous finding of the study is that the women who became pregnant since testing HIV positive had an increased risk of reporting pregnancy wastage. Gray et al. [2] demonstrated that spontaneous abortion is more common in HIV infected women in Uganda. Similarly, in a meta-analysis [3], the association between HIV positivity and the risk of spontaneous abortion ranged from 1.8 to 6 fold. Similarly, the association between HIV infection and increased risk of delivering low birth weight infants was studied in Tanzania [14], and associations between HIV infection and low birth weight, preterm delivery and intrauterine growth retardation were found. Gray et al. [2] and Brocklehurst and French [3] also showed that miscarriages, spontaneous abortions and stillbirths were more common in HIV infected women. A study to examine the effect of HIV infection on pregnancy wastage in Dar es Salaam, Tanzania [7] found that women presenting with spontaneous abortions had a significantly higher rate of HIV infection than those presenting for antenatal care or delivery, and that the HIV infected group had a much higher rate of recent history of fetal wastage (late abortions, neonatal deaths, premature delivery) than the non-infected group. In addition, HIV infected women delivered lower birth weight babies than the non-infected women [13].

Another important factor that is positively associated with the increased pregnancy wastage among HIV infected women is the duration of marriage. A large proportion of the respondents are married at a very young age with large age gap between the husband and the respondent. They are likely to be infected by their husbands, who engage in pre-marital as well as extra marital sexual encounters with female sex workers, which are quite common in the study area [15]. There is also sufficient evidence to show that HIV infected women tend to suffer from sexually transmitted infections (STIs), especially syphilis and in turn leading to spontaneous abortions [16]. Sexually transmissible diseases and medical complications were diagnosed almost twice as often among sero-positive women [17]. After controlling for factors such as sexually transmissible diseases, Leroy et al. [18], observed a significant increase in adverse obstetrical events, such as maternal postpartum haemorrhage and postoperative endometritis, as also confirmed by other authors [19].

The surprising finding is that the infected women living in joint families reported experiencing higher risk of pregnancy wastage. Stigma and discrimination from family members including parents and parents-in-law could be a probable reason as those experiencing stigma and discrimination are much less likely to receive care and support. Although the results are not presented here, 88 % of the women in our sample reported experiencing stigma and discrimination. Those experiencing stigma and discrimination are much less likely to access care and support as shown in a study in Vietnam, 60 % pregnant women refused HIV testing cited fear and discrimination as the dominant reason [20]. Both experiences of stigma and fears of stigma can have negative effects on health behavior and health outcomes. Stigma has been shown to be associated with psychological distress and with negative health outcomes [21], and has also been shown as a substantial barrier to uptake of HIV testing and other health services [22, 23, 24].

Most married women in India opt for tubectomy (female sterilization) within 3–5 years of marriage with one or more living children [25]. The longer duration of marriage, associated with increased reports of pregnancy wastage is indicative of poor access to permanent methods of contraception. The study shows that 6 % of the respondents are contributing to 62 % of the pregnancy wastage indicating multiple pregnancy wastages among a fewer number of women.

## Conclusion and implications

Those who were pregnant since testing HIV positive had about double the risk of reporting pregnancy wastage even after controlling the effect of other variables. As argued in the discussion, the prevalence of STIs among these women requires regular screening for STIs. In addition to this, high level of unmet need for family planning due to poor access to permanent (terminal) methods because of their HIV status, special attention needed for HIV infected women to provide spacing and permanent methods of contraceptive services. Similarly, to reduce stigma and discrimination, interventions including counselling, especially to the family members need to be introduced. A large proportion of the girls in the district continue to get married before the legal age at marriage, culturally appropriate communication programs that target unmarried adolescent girls and the people who influence the decisions should be introduced to increase womens’ age at marriage. Moreover, those girls who get married at younger ages are usually infected by their husbands who engage in pre and extra-marital sex with the female sex workers [14], and therefore, the programs needs to promote safe sex practices especially among the youth. Parents should also encourage HIV testing for their children before marriage.

### Limitations of the study

This study recruited a large sample of young women living with HIV, usually seen as a difficult population to interview. Since all or most of the PLHIVs in the district are registered with the community organization, *Chaitanya Mahila Sangha*, the sample of participants selected largely represent the young HIV infected women in the Bagalkot district. The study addressed an important reproductive aspect of pregnancy loss among a highly vulnerable and stigmatized population in a high HIV prevalent district in India. While this study has shown some very important findings, there are certain limitations.

- The study is a cross sectional and hence the profile of study participants refers to the time of survey instead of the profile at the time of pregnancy wastage occurred.
- This is not a randomized control trial and hence the risk of pregnancy wastage due to HIV positive status could have been different.
- Another limitation of the study could be associated with the sampling frame. As indicated by the HIV positive network, we believe that the sampling frame is complete. In case some HIV positive women register outside the district as a result of stigma and discrimination, then there could be bias in the outcome as a result of incomplete sampling frame.

## Abbreviations

HIV: Human Immuno Virus
AIDS: Acquired Immume Deficiency Syndrome
OR: Odds Ratio
AOR: Adjusted Odds Ratio
CI: Confidence Interval
STI: Sexually Transmitted Infection
CBO: Community Based Organization
SC/ST: Scheduled Tribe/Scheduled Caste
OBC: Other Backward Class
DIC+: Drop in center +
PPTCT: Prevention of parent to child transmission
